# Methods for the identification of farm escapees in feral mink (*Neovison vison*) populations

**DOI:** 10.1371/journal.pone.0224559

**Published:** 2019-11-11

**Authors:** Sussie Pagh, Cino Pertoldi, Heidi Huus Petersen, Trine Hammer Jensen, Mette Sif Hansen, Sussi Madsen, David Chr. Evar Kraft, Niels Iversen, Peter Roslev, Mariann Chriel

**Affiliations:** 1 Department of Chemistry and Bioscience—Section of Biology and Environmental Science, Aalborg University, Aalborg, Denmark; 2 Aalborg Zoo, Aalborg, Denmark; 3 National Veterinary Institute, Technical University of Denmark, Kgs, Lyngby, Denmark; 4 Department of Dentistry and Oral Health, University of Aarhus, Aarhus, Denmark; Wageningen Universiteit, NETHERLANDS

## Abstract

In Denmark, American mink (*Neovison vison*) have been bred for their fur since the mid-1920s. Mink escaping from farms may supply the feral population. Often, it is of biological and management interest to separate the population of feral mink (i.e. mink caught in the wild) in two groups: 1) mink born on farms i.e., escapees, and 2) mink born in the wild. In this study, two methods were used for separating feral mink into the two groups: a) Comparison of body length of farmed mink and feral mink, and b) Presence of a biomarker (tetracycline: an oral antibiotic used on mink farms). A total of 367 wild caught mink (from the mainland of Denmark and the island of Bornholm), and 147 mink from farms, collected during the period 2014–2018, were used for the analysis of body length. For the testing of tetracycline (TC) as a biomarker, 78 mink from farms where there was knowledge about TC treatment (with or without) were examined for fluorescent markings in the canine teeth. Results from both univariate analyses and Gaussian mixture model analysis demonstrated clear divisions between the mean body length (mean ± S.E., range) of farmed males (52.1 cm ± 0.4, 48–68) and farmed females (mean 44.0 ± 0.2, 40–50), and between farmed mink and wild caught mink. Mixture analysis identified two groups within each sex of the wild caught mink, one assigned to farmed mink (born in captivity) and another group of smaller mink suspected of being born in the wild. On Bornholm, the mean (±SD, range) length of males born in the wild was 43.7cm (± 0.3, 36–57) and for females 37.5cm (± 0.3, 32–45). The mean length (±SD, range) of males born in the wild in the mainland of Denmark was 42.5cm (± 2.3, 36–46) and for females 36.1cm (± 1.0, 34–37). Among the feral mink from mainland Denmark, 28.4% of males and 21.6% of females were identified as escapees, while 0% of the males and 1% of the females were identified as escapees among the wild caught mink on Bornholm. Eight percent of mink from farms using tetracycline were false negatives, while no false positives were found among mink from farms not using TC. TC fluorescence was found in five of 217 mink caught in the wild equivalent to 22% escapees in mainland Denmark. No TC markings were found in mink caught in the wild on Bornholm. In conclusion, both methods a) the body length of mink, and b) fluorescent biomarkers in canine teeth are considered as useful tools to identifing mink that have escaped from farms.

## Introduction

### Impact of invasive species

Both accidental and intentional introductions of alien species into nature may have a large impact on native ecosystems [1]. Although not all introduced species have an effect on the native biota, human mediated spread of species in general has led to homogenization and loss of biodiversity [1,2]. Species do not naturally have unlimited access to everywhere on earth due to physiological constraints, dispersal limitations and physical barriers in the landscape. However, human globalization has created new efficient dispersal routes for many species [1].

The American mink (*Neovison vison*), a medium sized semi-aquatic carnivorous mustelid native to north America, is an example of a species with human mediated spread in Europe. The American mink (here after mink, as European mink (*Mustela lutreola*) has never been recorded in Denmark) has during the past century spread to the Baltic countries, Scandinavia, the UK, Germany and Poland, with smaller populations in Holland, France, Spain and Italy [3]. In the overview of Bonesi and Palazon ground-nesting birds were the single-most mentioned group suffering from mink predation. In the UK and Belarus, a decline in the water vole (*Arvicola amphibius*) population was associated with the mink, for which the vole is a preferred prey [4,5]. Also, populations of the bank vole (*Clethrionomys glareolus*), and field vole (*Microtus agrestis*) on islands in Finland, declined significantly when the mink arrived and recovered when the mink were removed in a local eradication campaign [6]. When mink gain access to a rich food source e.g. crayfish, mink may be highly concentrated and more resilient in the face of control measures [7,8]. There are, however, a number of studies (among theses one from Denmark) that did not find any significant effect on prey species due to mink predation e.g. [8–10].

### Mink in Denmark

In Denmark, mink have been bred for their fur since the mid-1920s [11]. Currently, around 1,300 commercial mink farms house 3.4 million breeding dams (breeding females), resulting in approximately 17 million pelts per year. Thus, Denmark is one of the world’s largest producers of mink pelts [12]. Today, mink can be found in the wild all over Denmark due to farm escapes except from some small isolated islands [13]. Mink escape from farms both by accident and intentionally due to the opening of cages by animal rights activists. The annual number of mink escaping from Danish farms is unknown. However, according to a previous study, 80% of mink caught in the wild had recently escaped from farms, and showed a 25% chance of surviving the first three months in the wild [14]. It has therefor been debated on whether or not there is a true feral population in Denmark, like in some other European countries [14]. Danish farms may act as a source for the feral population, despite regulation in force regarding fencing the farm and traps within the farm area [15]. Regardless, of the existence of a true feral population of mink in Denmark or not, it is mandatory to reduce the propagule pressure (introduction of new individuals to the wild) of mink.

Since mink are regarded as invasive in Denmark, they can be culled all year round [16]. The annual hunting game bags of mink increased markedly from around 1000 mink in the late 1980s to around 8000 mink around the millennium [13,17]. Hereafter, bags have decreased, and today they are less than 2000 mink [18]. With emphasis on a relatively small feral population in Denmark, mink farm escapees may be a source of increasing genetic diversity and adaptation in local feral mink populations.

### Increasing body size in farmed mink

**Fig 1:**
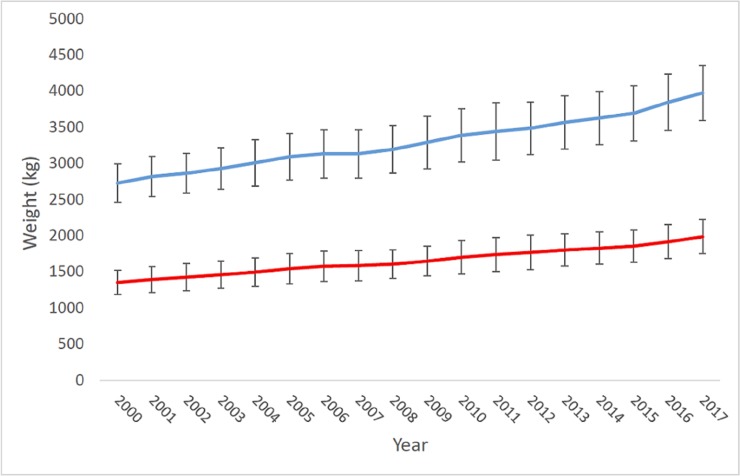
Increase in weight of breeding farm mink in Denmark from 2000 to 2017. (pers. comm. J. Clausen, Kopenhagen Fur).

Since the value of a mink pelt increases with the length and quality of the pelt, farmed mink are bred and fed to optimize these parameters. Mink bred on farms are selected for size, they have ample food and are kept in good health. If necessary, they are treated with antibiotics in case of disease e.g. diarrhoea or other illnesses. Accordingly, the size of farmed mink has increased over time. In 2007, less than 1% of the farmed male mink pelts from the Danish industry was between 101 and 107 cm, and no pelts were longer than 107 cm. In contrast, in 2018 23% of the male mink pelts were between 101 and 107 cm, and 8% were longer than 107 cm. Likewise, less than 1% of female pelts was between 83 and 89 cm and no female pelts were found in the category 89 to 95 cm in 2017. In 2018, 20% of female pelts were between 83 and 89 cm and 4% were between 89 and 95 cm (Jesper Clausen, Kopenhagen Fur, pers. comm.). Moreover, both breeding males and females have increased their mean weight by 70% for the past 10–15 years (Fig 1).

### Previous studies identifying escapees from farms

It is often of biological and management interest to be able to separate the feral mink population (i.e. mink caught in the wild) in two groups: 1) mink born on farms (i.e. escapees), and 2) mink born in the wild (i.e. wild mink). Studies of litter size and demography of the feral mink population may be biased by significant escapes of farmed individuals. According to a stochastic population simulation, the most influential parameters for the mink population in Denmark are mortality, fecundity and initial population size [17]. Also, immigration (including escapes from farms) will have an effect on the possibility to control a population [17].

In a previous Danish study of mink escapes based on microsatellite marking of 86 individuals and stable isotope analysis of 226 animals, more than 80% were found to have recently escaped from farms [19]. The isotope analysis was later questioned, as the isotope analysis is based on the assumption that feral mink mainly feed on a terrestrial diet, and that farmed mink mainly feed on a diet of marine origin. However, farmed mink are fed a mixture of by-products from both the fish and meat industries, and feral mink may feed on marine fish entering streams, and they are commonly found at harbours where they have access to marine fish.

In a model using skull size (condylobasal length and postorbital constrictions), it was possible to correctly classify the origin (farmed or wild) of 100% of male skulls and 90% of female skulls, hence it was concluded that the model should be effective for identifying farmed mink [20]. In a field application of this model, only one of 109 skulls collected in Ontario, Canada was identified as being of farm origin [20].

In a Polish study, chemical markers such as Hg and Cu were used for the identification of first generation mink farm escapees [21]. Analyses of the accumulation of 13 chemical elements in liver and kidney samples from farmed and wild mink showed significant differences in the levels of Hg and Cu between the two groups. The total Hg levels were up to 15-fold higher in the kidney, and up to 7-fold higher in the liver of wild mink compared to farmed mink [21].

Although these methods are successful in separating farmed mink from wild mink, the methods require both cleaning and measurements of skulls, or expensive analyses based on either genetics, isotopes or specific elements.

### Tetracycline in farmed mink and mink in the wild

**Fig 2:**
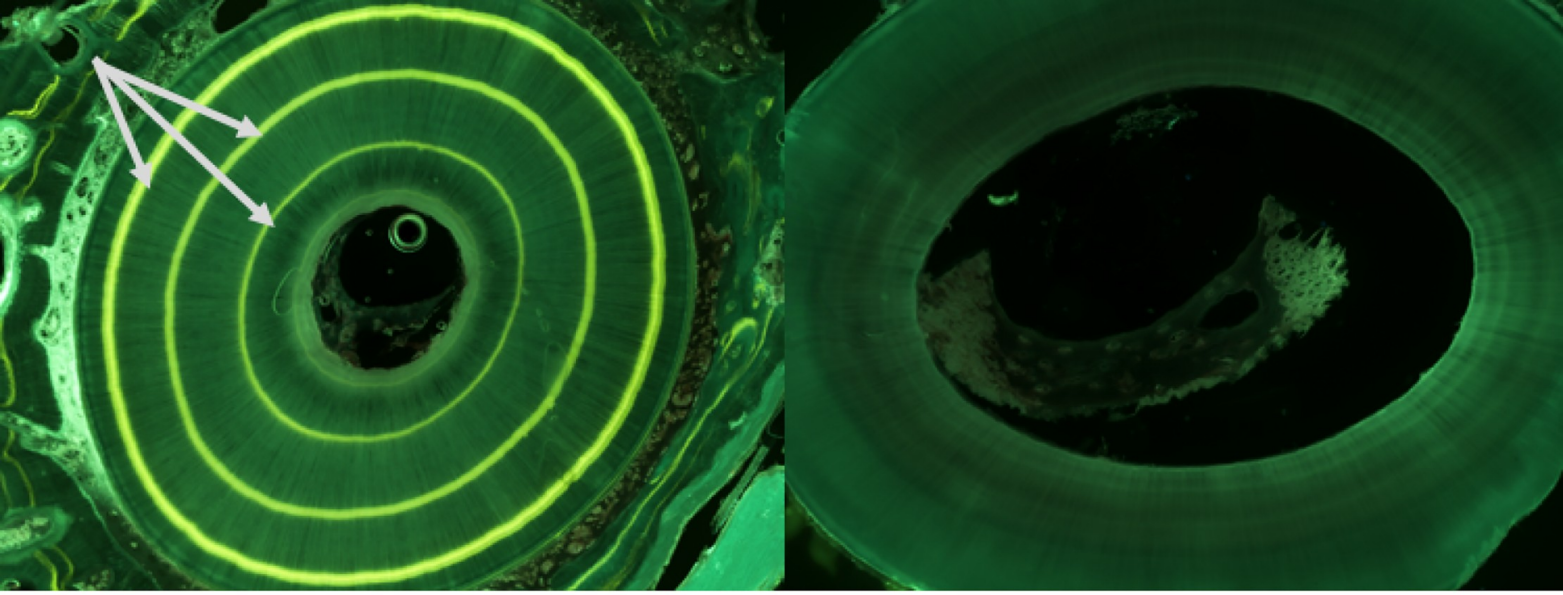
**Tooth root apex from mink with tetracycline (TC)(left) and without TC (right)**. Thin slices (70–100 μm) of the tooth root apex from mink under a microscope, left photo from a mink treated at least three times with tetracycline (TC) and right photo from an untreated mink. TC can be seen as bright yellow bands in the tooth cementum (arrows).

Tetracycline (TC) is a broad-spectrum antibiotic commonly used to treat Danish mink. TC is added to the feed (140g TC per ton feed) over a period of five days. In Denmark each mink gets around 200 g feed per day, hence a female mink weighing 1.5kg gets around 90mg TC per kg bodyweight per treatment. A fraction of the ingested TC is subsequently embedded in the growth layers of the dentin, dental cements and bone tissue, where it is detectable by epifluorescence microscopy for several years or for the rest of the animal´s life [22,23]. TC appears as yellow florescent markings (Fig 2). TC is widely used as biomarker in wildlife vaccination programs against viruses and parasites, and used in individual labeling in connection with "capture recapture" studies to estimate population sizes e.g. [24,25].

In Denmark TC is only used in husbandry and not in nature. Strict legislation against antibiotics in nature, prevents the presence of TC in wild animals. Hence, as TC is inaccessible to feral mink it can be used as a marker for farmed mink. TC for oral use was prescribed to 291 Danish mink farms (21% of 1385 farms in 2017) on average 1.8 times per prescribed mink farm in 2017, and to 180 prescribed farms in 2018 (Jan-Oct) on average 1.5 times per farm [26].

As controlling feral mink populations may be impeded by farm-animal escapes, a simple and unambiguous method to identify farmed mink in the feral mink population is of management interest. This will allow for appropriate actions to be taken.

The aim of this study is to test two methods distinguishing mink born on farms from wild born mink, which are easily feasible along with the general surveillance of mink: a) Difference in body length, and b) A biomarker (tetracycline) indicating a farmed mink.

## Material and methods

The study was based on 596 mink (449 wild caught) submitted for necropsy at the National Veterinary Institute from 2014–2018. Of these 367 wild caught and 147 farmed mink from the period September to May were used to identify farm escapes. Additionally, 50 mink raised on farms using TC and 28 mink from farms not using TC were collected.

Mink caught in the wild were submitted by hunters, while farmed mink originated from veterinary practitioners. Feral mink were sampled from Jutland, Funen, Zealand (considered as one group; mainland Denmark) and the island of Bornholm (treated as a single group due to its geographical distance from mainland Denmark).

The island of Bornholm (588 km^2^) is different from the mainland in terms of wild life. Bornholm is an island on rocky grounds, isolated from the mainland of Denmark by 143 km sea and from Sweden by 37 km. Wildlife differs in many ways from that of mainland Denmark, e.g. there are no native predators on the island. Therefore, collected mink from mainland Denmark and Bornholm are treated as different samples.

Until necropsy, the mink were stored at -20°C. At necropsy, sex and body length, to the nearest cm (from tip of the nose to the first vertebrate of the tail,) were recorded from all non-skinned mink.

### Mixture analyses of body size of farmed mink and mink caught in the wild

Gaussian mixture model analysis was used to separate farmed mink from wild born mink. For this analysis, the body length of 367 mink caught in the wild (133 from Bornholm and 234 from the mainland of Denmark) and 147 farmed mink were analysed. All individuals used in this analysis, were culled between September and May to ensure that only fully grown mink were included. Data from male (FM) and female (FF) farm mink were analysed separately. Wild mink were separated into four sub groups: Bornholm wild caught male (BWCM) and female (BWCF), and male (DWCM) and female (DWCF) mink caught in mainland Denmark. The number of individuals assigned to the above mentioned groups, and the means and the standard errors of mink body length excluding the tails were calculated for each group. There may be individuals not assigned to any group, therefore adding up the percentage of individuals assigned to the groups will not always added up to 100%. A box-plot was used to graphically depict the numerical data through their quartiles.

A one-way ANOVA and Tukey´s pairwise test were conducted to analyse differences in the mean body lengths of BWCM and BWCF, DWCM and DWCF, and FM and FF. If significant differences in means of body length were found between the groups, mixture analysis [27] was used in order to identify mink born on farms and mink born in the wild.

With mixture analysis the following was tested:

- Firstly, the relative number was quantified (expressed in %) of mink born on farms among mink caught in the wild on Bornholm, the number of BWCM and BWCF that were assigned to FM and FF, respectively, were analysed.
- Secondly, in order to quantify the relative number (expressed in %) of mink born on farms among mink caught in the wild in mainland Denmark, the number of DWCM and DWCF that were assigned to FM and FF, respectively, were calculated.

The mixture analysis was used to determine how many probable clusters (or groups) there could be recognised. Each set of mink (set 1: (FM + BCWM), set 2: (FF + BCWF), set 3: (FM + DCWM) and set 4: (FF + DCWF)) was divided into clusters using Gaussian mixture models, which evaluates the likelihood of a set consisting of a given number of clusters. A limit of maximum 5 clusters was used. For each number of clusters per set, the quality of the model was calculated using a corrected Akaike’s information criterion (AIC_C_).

A conservative estimate of mink assigned to born on farm, was obtained only assigning mink to the respective groups if the value of each probability density function was at least 20 times higher in one of the two groups. Hence, the lowest value of the probability density function was divided with the highest value. The value was expected to be highest for farm compared to the value for wild individuals. If the ratio was below 0.05, the mink was assigned to farm and therefore considered to be an escape.

The software program PAST was used for all statistical analysis (https://folk.uio.no/ohammer/past).

### Tetracycline as a biomarker

Two studies were conducted in order to test the usability of TC: 1) The canine teeth from 78 farmed mink, 50 raised on farms using TC and 28 mink from farms not using TC were tested for TC fluorescence. 2) The canine teeth of 217 wild caught mink (125 from the mainland Denmark and 92 from Bornholm) sampled in 2018 were tested for TC fluorescence by two experienced independend observers. All teeth were kept at -20°C until analysis.

Teeth from farmed mink and wild caught mink were tested for TC markings by using the same method. Canine teeth were fixed for 36 h in 4% w/v formaldehyde solution, dried overnight at 40°C, before being embedded in cold-polymerizing metylmethacrylate-based resin. 70–100 μm thick saw sections were hereafter made 2 and 3 mm from the apex of the root of the tooth with a Leiden saw (Meprotech, Holland Heerhugowaard). The unstained saw sections were examined using a fully automated Olympus BX61 microscope (Olympus Ltd, Ballerup, Denmark) equipped with a DP80 camera (Olympus Ltd, Ballerup, Denmark). Two TC excitation (Ex) and emission (Em) filter sets were used for evaluating TC markings (set 1: Ex400-440 nm; Em475 nm/long-pass and set 2: Ex385-425 nm; Em520-580nm). False negatives are defined as lack of TC markings in mink teeth from farms treating mink with TC, false positives are defined as yellow markings in mink teeth from farms not treating their mink with TC.

## Results

### Mean body length of different groups of mink

The mean body length of BWCM, BWCF, DWCM, DWCF, and FM and FF were significantly different (Table 1) (F_5,508_ = 189.8, p < 0.001). The results from Tukey’s pairwise test are shown in Table 2.

**Table 1:** Mink body length (cm) excluding the tails of farm males (FM), farmed females (FF), Bornholm wild caught males (BWCM) and females (BWCF), mainland Denmark wild caught males (DWCM) and females (DWCF) from samples collected from 2014–2018. Sample size (N), mean, minimum (Min), maximum (Max) values, standard errors (std. error), median and 25 and 75 percentiles (prcntil).

	FM	FF	BWCM	BWCF	DWCM	DWCF
N	63	84	84	49	95	139
Min	48	40	36	32	36	34
Max	68	50	57	45	52	49
Mean	52.1	44.0	43.7	37.5	44.1	40.7
Std. error	0.4	0.2	0.3	0.3	0.4	0.3
Median	52	44	44	38	44	41
25 prcntil	51	43	42	36	42	38
75 prcntil	53	45	45	39	47	43

**Table 2:** Tukey’s pairwise test was conducted to test for significant differences in mean body length excluding the tails of farm males (FM), farmed females (FF), Bornholm wild caught males (BWCM) and females (BWCF), mainland Denmark wild caught males (DWCM) and females (DWCF) (*** = p < 0.0001; n.s. = non-significant) from samples collected from 2014–2018.

	FM	FF	BWCM	BWCF	DWCM	DWCF
FM		***	***	***	***	***
FF			n.s.	***	n.s.	***
BWCM				***	n.s.	***
BWCF					***	***
DWCM						***
DWCF						

**Fig 3:**
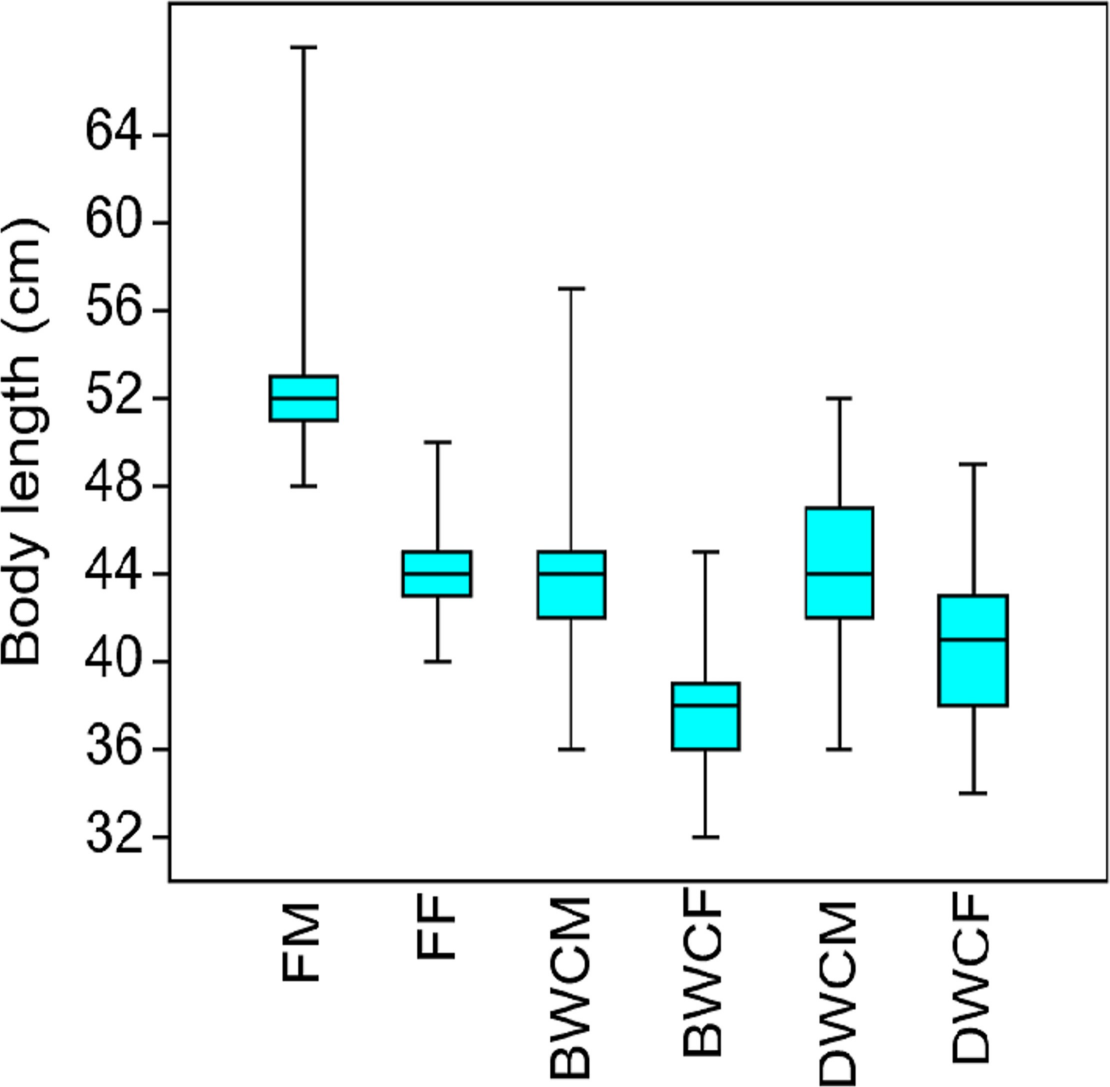
Box plot of the mink body length. Length excluding the tails of farm males (FM), farmed females (FF), Bornholm wild caught males (BWCM) and females (BWCF), from samples collected from 2014–2018. Denmark mainland wild caught males (DWCM) and females (DWCF). The rectangles represent the 95% confidence intervals.

The box plot (Fig 3) shows that both farmed males and males born in the wild are statistically larger than the respective females (p<0.005). The largest sexual dimorphism was observed for farmed mink. Farmed males are significantly larger than males from Bornholm and males from the remaining Denmark (Fig 3). Farmed females are significantly larger than females born in the wild on Bornholm and wild born females from mainland Denmark (Fig 3).

The Tukey’s tests revealed that males were significantly larger than females. Moreover, FM were significantly (p<0.05) longer than the BWCM and DWCM (Table 2). Also, the FF were significantly longer than the BWCF and DWCF (p< 0.05). Finally, DWCF were significantly longer than BWCF i.e. the sexual dimorphism of feral mink on Bornholm was larger than in the restt of Denmark (Table 2).

**Fig 4:**
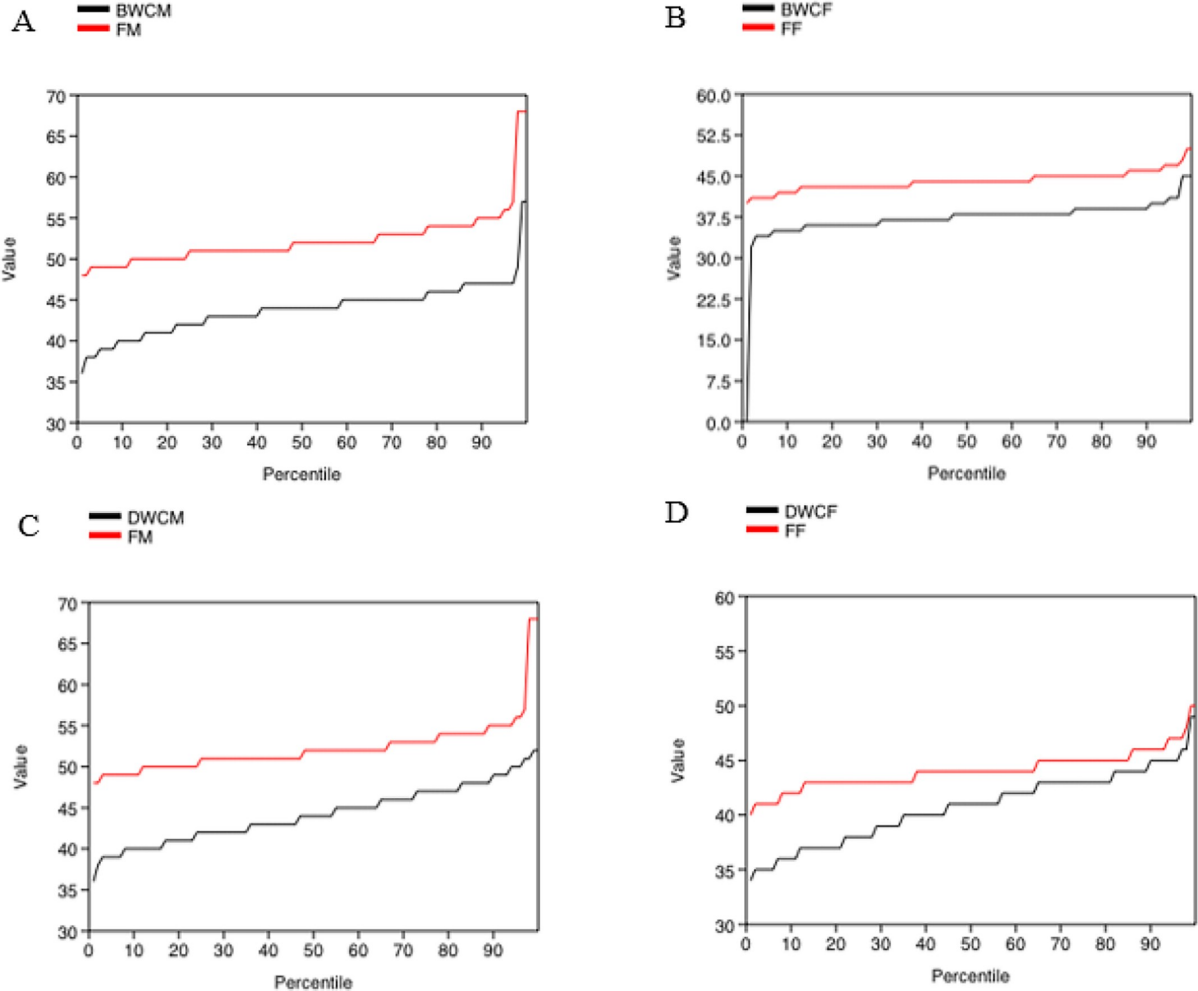
a-d. Cumulative curves of body length of farm mink and mink caught in the wild in Denmark from 2014–2018.

Also, cumulative curves demonstrate clear divisions in the body length of farmed mink and mink caught in the wild (Fig 4A–4C).

### Groups identified by mixture analysis

In all the sets analyzed by mixture analysis, the optimal number of clusters was two (assigned to two normal distributions). Hence, the number of individuals could be assigned to cluster (1) or cluster (2). A mean and standard deviation characterizes the distributions of the two clusters, and the number of individuals in % of the clusters is provided by the software.

Considering that the individuals that are assigned to cluster 1) are the farm mink and that individuals assigned to cluster 2) are the mink born in the wild, the following sets are analyzed: set 1: (FM + BCWM), set 2: (FF + BCWF), set 3:(FM + DWCM), set 4: (FF + DWCF).

The mixture analysis of set 1 identifies two clusters, one with a body length of mean ± SD 43.06 ± 2.6 cm (BWCM), and one group with a mean ± SD of 50.41 ± 4.0 cm (FM). Of the 84 wild caught males, 72 (85.7%) were assigned to mink born in the wild, however, with a more conservative criterion demanding a 0.05 criterion, no male mink from Bornholm were assigned to the farm cluster. This means that the BWCM population can be considered as a truly feral population, i.e. 0% of the male mink caught on Bornholm is considered to be escapees (Table 1, S1 Table, S2 Table).

Likewise, the mixture analysis of set 2 identified two natural groups. Of the 49 wild caught females on Bornholm, 48 (97.9%) were identified as BWCF, i.e. truly feral. Only one female caught in the wild on Bornholm was assigned to the farm cluster. Following the criterion 0.05, the same result was obtained, hence approx. 1% of the females captured in the wild on Bornholm can be considered escapees (Table 1, S1 Table, S2 Table).

The mixture analysis of set 3 identifies two clusters. Of the 95 wild caught males in the mainland of Denmark, 44 individuals (46.3%) were identified as DWCM, i.e. truly feral. Following the criterion 0.05 twenty-seven males captured in the wild were considered as escapees, which means that approx. 28.4% of the males captured in the wild in mainland Denmark can be considered escapees (Table 1, S1 Table, S2 Table).

The mixture analysis set 4 identifies two clusters. Of the 139 wild caught females in the mainland of Denmark, 48 (34.5%) were identified as DWCF, i.e. truly feral. Following the criterion 0.05, thirty females captured in the wild were considered as escapees, which means that approx. 21.6% of the females captured in the wild can be considered escapees (Table 1, S1 Table, S2 Table).

On Bornholm, the mean (±SD, range) length of males born in the wild was 43.7cm (± 0.3, 36–57) and for females 37.5cm (± 0.3, 32–45). The mean length (±SD, range) of males born in the wild in the mainland of Denmark was 42.5cm (± 2.3, 36–46) and for females 36.1cm (± 1.0, 34–37).

### Tetracycline as a biomarker

In the study of TC in farmed mink, 46 of the 50 mink teeth were positive for TC fluorescence (92.0%) from the farms using TC, i.e. four false negative were found. No TC fluorescence was found in the teeth of the 28 mink from the control farms not using TC, i.e., no false positives were detected.

Of the 125 wild caught mink tested for TC fluorescence from mainland Denmark five (4%) were TC positive, while no TC positive from Bornholm were found. Bearing in mind that only 21% of the mink farms use TC and that 8% of these are false negatives, this suggests that around 21% (4% multiplied by 100/21*1.08) of the mink on the mainland of Denmark are likely escapees.

## Discussion

### Body length as a method of identifying escapees

Results from the univariate analysis of body length demonstrated that male and female farmed mink are significantly longer than the feral mink population. The mixture analysis demonstrated that it is possible to recognise two groups in the wild caught population and separate wild living mink from escaped farmed mink. The mixture analysis of wild caught mink from Bornholm show that there is a wild living feral population on Bornholm, almost without recent escapees. No male mink and 1% of female mink are identified as escapees among the feral mink on Bornholm. Hence, there are either few mink escapees from farms on Bornholm or a very low survival of mink escaped from farms. The mixture analysis of mink from the remaining part of Denmark indicates that there are more escapees or better survival of mink from farms in these populations; 28.4% escapees of males and 21.6% of females.

### Tetracycline as a biomarker to identify escapees

Tetracycline fluorescent markings was highly detectable in mink from farms using TC (92.0% TC positive). However four false negatives (8%) were found. The false negatives may be due to four mink not receiving sufficient amounts of TC to fluorescently mark bone tissue and teeth, e.g. if they did not eat enough feed. No TC positive teeth were found in the mink from the control farms not using TC. This indicates that TC fluorescence could be a simple and convenient indicator for escapees. However, TC or related compounds have to be given to all farmed mink (or the majority), in order for them to be useful tools in field studies. TC is an antimicrobial agent and is only allowed to be used for treating diseased animals. Therefore, the use of biomarkers other than TC must be considered in future studies. Dosing of appropriate biomarkers to mink feed could then provide further information about the capability of mink born on farms to survive and spread in the wild. The TC markings in the wild caught mink suggested that approximately 21% of wild caught mink are escapees in the mainland of Denmark, while there are no apparent escapees on Bornholm. The TC markings are therefore comparable with the number of escapees found using mixture analysis.

### Survival of escapees in the wild

An increasing number of papers support the hypotheses that geographic variation in body size within a species is caused by local or temporal variation in food supply (e.g. [28–33]. Basically, there are two ways that food influences the body size of fully grown individuals. First, ample nutrition during the individual’s development is essential in obtaining optimal body weight. In free ranging populations, variation in food supply from year to year is known to produce cohorts of generations with different mean body weight caused by yearly fluctuations in food supply [33–35]. Secondly, the fully grown body size of an individual may genetically be adapted to “bottleneck” periods, e.g. winter periods with sparse food supply. Individuals with relatively small body size need less food in “bottleneck” periods to maintain energy reserves and therefore have larger chances of surviving than large individuals do [36]. On the other hand, a small body size may lead to a relatively low reproduction. Hence, body size is a balance between advantages and disadvantages, e.g. energy need and reproduction [31].

Some introduced species are known to change rapidly during one or a few generations in response to a changing environment [37]. This also applies to mink [31][38,39]. In a survey of the body size of mink during their colonization of Warta Mouth National Park, west Poland, the body size of mink changed significantly from 1996 to 2004 [31]. The mean body weight of males dropped 13% from 1.36 to 1.18 kg and that of females dropped 16% from 0.83 to 0.70 kg [31]. These changes were ascribed to changes in food availability [31].

The natural selection pressure is released in farmed mink [40]. Thus, farmed mink are poorly adapted to natural conditions [14]. Natural selection on farmed mink is therefore expected to be strong immediately after escape from a farm. Generations of mink living in the wild have to adapt their body size, colour, behaviour and biology to be able to survive under natural conditions [31]. Mink escaped from Danish farms have previously shown a 25% chance of surviving during the first three months in the wild [14]. This has led to a debate on whether or not there is a true feral population in Denmark. The results of our study where we identified a sub-population of smaller feral mink strongly indicate that there is a feral population of mink in Danish nature adapted to natural conditions. The absence of native predators on Bornholm [13], may allow a larger sexual dimorphism between males and females on the island [41–43].

## Concluding remarks

In management plans as well as in studies of reproduction, demography, diet and health of feral mink, it is essential to be able to separate mink born on farms from mink born in the wild, to prevent bias of the results due to different life conditions between farmed mink and feral mink. Both measurements of body length of mink caught in the wild and biomarkers added to the food of mink at farms are considered as useful tools to separate farmed mink from wild born mink. However, biomarkers have to be given to all farmed mink or a known and significant fraction in order to be useful tools in field studies. Therefore, the use of biomarkers other than TC must be considered in future studies. Using biomarkers in the feed each year, will also provide information about the capability of mink born on farms to survive in the wild.

## Supporting information

S1 Table Mixture analysis of four sets of farmed and wild caught mink. Wild mink were separated into four sub groups: Bornholm wild caught male (BWCM) and female (BWCF), and male (DWCM) and female (DWCF) mink caught in mainland Denmark. Farmed mink were separated into females (FF) and males (FM).
(PDF)

S2 Table Data used in the mixture analysis. (PDF)
