# Supplementary material for: Methods for the identification of farm escapees in feral mink (Neovison vison) populations
Source: PLoS One. 2019 Nov 11;14(11):e0224559. doi: 10.1371/journal.pone.0224559 (PMC6852605; doi:10.1371/journal.pone.0224559)
Supplement: S1 Table — Wild mink were separated into four sub groups: Bornholm wild caught male (BWCM) and female (BWCF), and male (DWCM) and female (DWCF) mink caught in mainland Denmark. Farmed mink were separated into females (FF) and males (FM). (PDF) [file pone.0224559.s001.pdf]

S1 Table

Mixture analysis on set 1:(FM + BCWM),				
Prob		Mean	Stdev	correctly assigned (%)
Cluster 1 FM	0.58	50.41	04.00	63/63 correctly assigned = 100%
Cluster 2 BWCM	0.42	43.06	02.06	72/84 correctly assigned = 85.7%, (criterion 0.05 = 0 escapees)
set 2: (FF + BCWF),				
Prob		Mean	Stdev	correctly assigned (%)
Cluster 1 FF	0.62174	44.13	01.06	83/84 correctly assigned = 98.8%
Cluster 2 BWCF	0,37826	37.49	01.09	48/49 correctly assigned = 97.9% (criterion 0.05 = 1 escapee).
set 3:(FM + DWCM),				
Prob.		Mean	Stdev	correctly assigned (%)
Cluster 1 FM	0,73	49.40	4,0,	63/63 correctly assigned = 100%
Cluster 2 DWCM	0,27	41.60	02.00	44/95 correctly assigned = 46.32%, (criterion 0.05 = 27 escapees).
set 4: (FF + DWCF)				
Prob		Mean	Stdev	correctly assigned (%)
Cluster 1 FF		43.18	02.00	84/84 correctly assigned = 100%
Cluster 2 DWCF		36.99	01.05	48/139 correctly assigned = 34.5% (criterion 0.05 = 30 escapees).
