# Supplementary material for: Methods for the identification of farm escapees in feral mink (Neovison vison) populations
Source: PLoS One. 2019 Nov 11;14(11):e0224559. doi: 10.1371/journal.pone.0224559 (PMC6852605; doi:10.1371/journal.pone.0224559)
Supplement: S2 Table — (PDF) [file pone.0224559.s002.pdf]

S2 Table. Mixture analysis data.

Group	Collection year	Death date	Body length
BWCM1	2015	29-01-2015	47
BWCM2	2015	17-10-2014	40
BWCM3	2015	16-11-2014	44
BWCM4	2015	24-09-2014	40
BWCM5	2015	10-02-2015	45
BWCM6	2015	09-03-2015	47
BWCM7	2015	12-04-2015	46
BWCM8	2015	08-02-2015	45
BWCM9	2015	22-09-2014	44
BWCM10	2015	30-09-2014	43
BWCM11	2015	14-09-2014	41
BWCM12	2015	08-02-2015	44
BWCM13	2015	15-04-2015	43
BWCM14	2015	28-03-2015	46
BWCM15	2015	02-12-2014	44
BWCM16	2015	12-10-2014	38
BWCM17	2015	06-03-2015	45
BWCM18	2015	03-02-2015	47
BWCM19	2016	18-10-2015	57
BWCM20	2016	06-10-2015	44
BWCM21	2016	04-10-2015	49
BWCM22	2016	19-09-2015	42
BWCM23	2016	23-12-2015	42
BWCM24	2016	09-09-2015	38
BWCM25	2016	14-12-2015	47
BWCM26	2016	11-09-2015	44
BWCM27	2016	02-12-2015	45
BWCM28	2016	11-12-2015	45
BWCM29	2016	08-09-2015	45
BWCM30	2016	22-11-2015	47
BWCM31	2016	12-03-2016	41
BWCM32	2016	28-03-2016	43
BWCM33	2016	08-03-2016	45
BWCM34	2016	11-02-2016	39
BWCM35	2016	28-03-2016	41
BWCM36	2016	18-03-2016	46
BWCM37	2016	10-02-2016	45
BWCM38	2016	28-01-2016	44
BWCM39	2016	12-03-2016	45
BWCM40	2016	28-03-2016	42
BWCM41	2017	23-12-2016	44
BWCM42	2017	06-04-2017	43
BWCM43	2018	16-09-2017	45
BWCM44	2018	27-12-2017	47
BWCM45	2018	13-02-2018	44
BWCM46	2018	25-09-2018	36
BWCM47	2018	02-02-2018	44
BWCM48	2018	19-01-2018	46
BWCM49	2018	24-03-2018	39
BWCM50	2018	19-03-2018	47
BWCM51	2018	25-09-2018	39
BWCM52	2018	06-09-2018	43
BWCM53	2018	27-11-2017	46
BWCM54	2018	09-09-2018	42
BWCM55	2018	27-09-2018	44
BWCM56	2018	04-12-2018	44

BWCM57	2018	23-09-2018	44
BWCM58	2018	26-09-2018	40
BWCM59	2018	20-09-2018	42
BWCM60	2018	05-04-2018	44
BWCM61	2018	30-01-2018	40
BWCM62	2018	26-02-2018	43
BWCM63	2018	18-01-2018	47
BWCM64	2018	12-03-2018	46
BWCM65	2018	17-09-2018	43
BWCM66	2018	28-09-2018	40
BWCM67	2018	04-05-2018	47
BWCM68	2018	16-01-2018	43
BWCM69	2018	01-09-2018	39
BWCM70	2018	15-10-2018	45
BWCM71	2018	06-09-2018	41
BWCM72	2018	27-12-2017	43
BWCM73	2018	01-02-2018	43
BWCM74	2018	14-07-2018	45
BWCM75	2018	06-09-2018	47
BWCM76	2018	14-09-2018	42
BWCM77	2018	25-03-2018	45
BWCM78	2018	13-04-2018	45
BWCM79	2018	17-09-2018	41
BWCM80	2018	28-01-2018	45
BWCM81	2018	14-09-2018	44
BWCM82	2018	24-01-2018	41
BWCM83	2018	21-03-2018	46
BWCM84	2018	24-03-2018	45
BWCF1	2015	07-09-2015	35
BWCF2	2015	06-01-2015	41
BWCF3	2015	11-03-2015	39
BWCF4	2015	24-02-2015	38
BWCF5	2015	24-02-2015	38
BWCF6	2015	05-09-2014	38
BWCF7	2015	09-03-2015	36
BWCF8	2015	27-01-2015	32
BWCF9	2016	01-02-2016	38
BWCF10	2016	02-10-2015	38
BWCF11	2016	11-09-2015	36
BWCF12	2016	02-11-2015	37
BWCF13	2016	11-12-2015	36
BWCF14	2016	04-11-2015	37
BWCF15	2016	22-09-2015	39
BWCF16	2016	09-09-2015	45
BWCF17	2016	05-11-2015	38
BWCF18	2016	10-09-2015	34
BWCF19	2016	09-10-2015	38
BWCF20	2016	14-10-2015	37
BWCF21	2016	17-09-2015	36
BWCF22	2016	09-09-2015	38
BWCF23	2016	05-09-2015	39
BWCF24	2016	14-09-2015	37
BWCF25	2016	18-01-2016	36
BWCF26	2016	03-02-2016	39
BWCF27	2016	11-03-2016	37
BWCF28	2016	14-03-2016	39
BWCF29	2016	14-05-2016	38
BWCF30	2016	26-03-2016	38
BWCF31	2016	06-04-2016	40

BWCF32	2017	05-10-2016	38
BWCF33	2017	21-07-2017	35
BWCF34	2018	24-08-2018	40
BWCF35	2018	24-09-2018	38
BWCF36	2018	20-09-2018	34
BWCF37	2018	13-09-2018	36
BWCF38	2018	31-10-2018	39
BWCF39	2018	20-03-2018	37
BWCF40	2018	14-08-2018	35
BWCF41	2018	03-11-2018	38
BWCF42	2018	15-09-2018	36
BWCF43	2018	15-10-2018	37
BWCF44	2018	29-10-2018	39
BWCF45	2018	23-10-2017	39
BWCF46	2018	25-09-2018	37
BWCF47	2018	02-10-2018	39
BWCF48	2018	01-10-2018	36
BWCF49	2018	03-09-2018	36
DWCM1	2015	22-09-2015	42
DWCM2	2015	25-09-2015	45
DWCM3	2015	01-09-2014	40
DWCM4	2015	02-12-2013	44
DWCM5	2015	19-10-2013	41
DWCM6	2015	13-09-2014	46
DWCM7	2015	06-11-2012	42
DWCM8	2015	07-09-2014	43
DWCM9	2015	14-09-2014	40
DWCM10	2015	15-09-2014	40
DWCM11	2015	08-09-2014	45
DWCM12	2015	04-09-2014	39
DWCM13	2015	03-09-2014	41
DWCM14	2015	01-11-2014	41
DWCM15	2015	15-09-2014	43
DWCM16	2015	31-03-2015	46
DWCM17	2015	28-04-2008	42
DWCM18	2016	01-11-2015	49
DWCM19	2016	01-11-2015	51
DWCM20	2016	27-08-2015	49
DWCM21	2016	28-12-2015	42
DWCM22	2016	15-10-2015	39
DWCM23	2016	02-11-2015	44
DWCM24	2016	11-12-2015	45
DWCM25	2016	07-02-2016	48
DWCM26	2016	17-02-2016	43
DWCM27	2016	26-01-2016	46
DWCM28	2016	09-12-2014	42
DWCM29	2016	01-05-2016	40
DWCM30	2016	16-02-2016	48
DWCM31	2016	29-11-2015	46
DWCM32	2017	19-03-2017	48
DWCM33	2017	03-10-2016	44
DWCM34	2017	06-12-2016	47
DWCM35	2017	05-04-2017	48
DWCM36	2017	20-09-2016	44
DWCM37	2017	27-09-2016	46
DWCM38	2017	28-03-2017	43
DWCM39	2017	05-10-2017	52
DWCM40	2017	20-03-2017	48
DWCM41	2017	20-03-2017	47

DWCM42	2018	11-09-2017	50
DWCM43	2018	15-12-2017	42
DWCM44	2018	15-10-2017	43
DWCM45	2018	19-03-2018	50
DWCM46	2018	19-03-2018	47
DWCM47	2018	19-03-2018	49
DWCM48	2018	19-03-2018	47
DWCM49	2018	19-03-2018	50
DWCM50	2018	19-03-2018	49
DWCM51	2018	01-01-2017	39
DWCM52	2018	01-01-2017	44
DWCM53	2018	01-01-2017	47
DWCM54	2018	18-11-2017	44
DWCM55	2018	01-03-2018	47
DWCM56	2018	26-09-2018	46
DWCM57	2018	28-10-2018	39
DWCM58	2018	03-10-2018	41
DWCM59	2018	07-03-2018	43
DWCM60	2018	07-02-2018	46
DWCM61	2018	30-01-2018	45
DWCM62	2018	22-11-2017	45
DWCM63	2018	01-12-2016	40
DWCM64	2018	20-01-2018	51
DWCM65	2018	27-08-2017	42
DWCM66	2018	22-11-2017	45
DWCM67	2018	13-03-2017	43
DWCM68	2018	03-12-2016	41
DWCM69	2018	03-11-2016	42
DWCM70	2018	17-09-2017	40
DWCM71	2018	27-09-2017	47
DWCM72	2018	15-11-2016	40
DWCM73	2018	14-12-2016	41
DWCM74	2018	07-10-2016	47
DWCM75	2018	02-09-2016	42
DWCM76	2018	01-09-2016	48
DWCM77	2018	20-01-2017	45
DWCM78	2018	02-10-2014	38
DWCM79	2018	07-10-2015	36
DWCM80	2018	25-09-2017	43
DWCM81	2018	20-09-2017	45
DWCM82	2018	10-09-2016	43
DWCM83	2018	01-06-2017	39
DWCM84	2018	12-12-2016	42
DWCM85	2018	14-04-2018	42
DWCM86	2018	25-10-2018	42
DWCM87	2018	29-09-2018	44
DWCM88	2018	08-09-2018	44
DWCM89	2018	27-09-2017	47
DWCM90	2018	15-11-2016	40
DWCM91	2018	11-03-2018	47
DWCM92	2018	29-01-2018	41
DWCM93	2018	22-03-2018	46
DWCM94	2018	27-01-2018	45
DWCM95	2018	15-01-2018	43
DWCF1	2014	01-12-2013	40
DWCF2	2015	01-11-2014	41
DWCF3	2015	12-03-2015	43
DWCF4	2015	03-03-2015	42
DWCF5	2015	25-11-2014	43

DWCF6	2015	01-05-2015	37
DWCF7	2015	12-04-2015	40
DWCF8	2015	21-02-2015	41
DWCF9	2015	01-12-2014	43
DWCF10	2015	22-09-2015	46
DWCF11	2015	05-10-2015	43
DWCF12	2015	16-09-2014	41
DWCF13	2015	21-09-2015	43
DWCF14	2015	21-11-2014	37
DWCF15	2015	14-10-2015	42
DWCF16	2015	09-12-2013	40
DWCF17	2015	25-09-2013	45
DWCF18	2015	17-12-2015	44
DWCF19	2015	26-09-2015	42
DWCF20	2015	01-09-2014	35
DWCF21	2015	16-09-2014	35
DWCF22	2015	07-09-2014	37
DWCF23	2015	14-09-2014	36
DWCF24	2015	02-09-2014	38
DWCF25	2015	04-09-2014	40
DWCF26	2015	02-09-2014	40
DWCF27	2015	05-03-2015	41
DWCF28	2015	01-01-2015	37
DWCF29	2015	01-01-2015	42
DWCF30	2016	26-11-2015	46
DWCF31	2016	02-01-2016	49
DWCF32	2016	26-10-2016	43
DWCF33	2016	13-01-2016	41
DWCF34	2016	12-11-2015	44
DWCF35	2016	14-11-2015	38
DWCF36	2016	18-02-2016	43
DWCF37	2016	02-02-2016	45
DWCF38	2016	17-10-2015	43
DWCF39	2016	23-05-2016	41
DWCF40	2016	03-12-2015	44
DWCF41	2016	12-10-2016	42
DWCF42	2016	25-11-2015	41
DWCF43	2016	04-01-2016	44
DWCF44	2016	12-01-2016	40
DWCF45	2016	05-01-2016	43
DWCF46	2016	10-09-2016	41
DWCF47	2016	10-10-2016	37
DWCF48	2016	26-09-2016	37
DWCF49	2016	02-11-2015	41
DWCF50	2016	13-11-2015	41
DWCF51	2016	10-09-2015	41
DWCF52	2016	27-10-2014	37
DWCF53	2016	02-10-2014	38
DWCF54	2016	29-01-2016	37
DWCF55	2016	10-02-2016	37
DWCF56	2016	18-08-2016	39
DWCF57	2016	02-04-2016	37
DWCF58	2016	28-03-2016	39
DWCF59	2016	05-04-2016	39
DWCF60	2017	28-09-2016	44
DWCF61	2017	20-11-2016	43
DWCF62	2017	17-12-2016	41
DWCF63	2017	21-02-2008	38
DWCF64	2017	24-10-2017	43

DWCF65	2017	19-11-2016	43
DWCF66	2017	12-11-2014	44
DWCF67	2017	21-03-2014	45
DWCF68	2017	24-01-2017	44
DWCF69	2017	19-06-2017	42
DWCF70	2017	04-11-2017	45
DWCF71	2017	04-11-2017	45
DWCF72	2017	05-10-2017	45
DWCF73	2017	05-10-2017	43
DWCF74	2018	03-12-2017	40
DWCF75	2014	01-12-2013	40
DWCF76	2018	19-03-2018	39
DWCF77	2018	19-03-2018	43
DWCF78	2018	19-03-2018	40
DWCF79	2018	19-03-2018	42
DWCF80	2018	01-01-2017	41
DWCF81	2018	01-01-2017	45
DWCF82	2018	03-12-2017	40
DWCF83	2018	19-10-2017	40
DWCF84	2018	16-03-2018	44
DWCF85	2018	11-03-2018	44
DWCF86	2018	19-12-2017	43
DWCF87	2018	09-09-2014	41
DWCF88	2018	01-11-2017	44
DWCF89	2018	09-11-2018	43
DWCF90	2018	08-10-2018	45
DWCF91	2018	05-06-2018	45
DWCF92	2018	26-09-2018	43
DWCF93	2018	27-09-2018	43
DWCF94	2018	06-09-2018	41
DWCF95	2018	15-04-2018	45
DWCF96	2018	27-03-2018	43
DWCF97	2018	23-10-2018	44
DWCF98	2018	23-10-2018	43
DWCF99	2018	10-10-2018	43
DWCF100	2018	08-09-2018	42
DWCF101	2018	01-10-2018	42
DWCF102	2018	05-09-2018	41
DWCF103	2018	13-11-2018	43
DWCF104	2018	26-09-2018	36
DWCF105	2018	30-10-2018	36
DWCF106	2018	20-04-1918	42
DWCF107	2018	19-01-2018	47
DWCF108	2018	25-01-2018	42
DWCF109	2018	20-09-2018	39
DWCF110	2018	01-12-2016	36
DWCF111	2018	01-12-2016	38
DWCF112	2018	01-12-2016	35
DWCF113	2018	01-12-2016	38
DWCF114	2018	22-01-2017	38
DWCF115	2018	12-10-2016	40
DWCF116	2018	20-09-2016	36
DWCF117	2018	22-10-2016	37
DWCF118	2018	01-09-2017	37
DWCF119	2018	16-11-2016	36
DWCF120	2018	08-12-2016	39
DWCF121	2018	16-12-2016	38
DWCF122	2018	05-11-2016	38
DWCF123	2018	14-12-2016	35

DWCF124	2018	01-04-2017	40
DWCF125	2018	26-09-2016	39
DWCF126	2018	26-03-2017	34
DWCF127	2018	07-10-2016	40
DWCF128	2018	18-12-2017	35
DWCF129	2018	08-09-2017	37
DWCF130	2018	02-04-2018	38
DWCF131	2018	11-09-2018	36
DWCF132	2018	26-10-2018	35
DWCF133	2018	26-09-2016	39
DWCF134	2018	26-03-2017	34
DWCF135	2018	14-10-2018	42
DWCF136	2018	20-10-2017	36
DWCF137	2018	04-10-2017	37
DWCF138	2018	01-04-2017	43
DWCF139	2018	01-04-2017	43
FM1	2018	16-03-2018	52
FM2	2018	16-03-2018	57
FM3	2018	16-03-2018	56
FM4	2018	19-03-2018	53
FM5	2018	21-03-2018	50
FM6	2018	21-03-2018	51
FM7	2018	21-03-2018	51
FM8	2018	21-03-2018	54
FM9	2018	21-03-2018	52
FM10	2018	21-03-2018	51
FM11	2018	21-03-2018	51
FM12	2018	21-03-2018	51
FM13	2018	21-03-2018	52
FM14	2018	21-03-2018	51
FM15	2018	21-03-2018	55
FM16	2018	21-03-2018	51
FM17	2018	21-03-2018	53
FM18	2018	21-03-2018	54
FM19	2018	21-03-2018	52
FM20	2018	21-03-2018	53
FM21	2018	21-03-2018	50
FM22	2018	21-03-2018	53
FM23	2018	21-03-2018	51
FM24	2018	21-03-2018	51
FM25	2018	21-03-2018	50
FM26	2018	21-03-2018	50
FM27	2018	21-03-2018	51
FM28	2018	21-03-2018	51
FM29	2018	21-03-2018	49
FM30	2018	21-03-2018	50
FM31	2018	21-03-2018	52
FM32	2018	21-03-2018	53
FM33	2018	21-03-2018	52
FM34	2018	21-03-2018	51
FM35	2018	21-03-2018	52
FM36	2018	21-03-2018	54
FM37	2018	21-03-2018	52
FM38	2018	21-03-2018	52
FM39	2018	21-03-2018	49
FM40	2018	21-03-2018	51
FM41	2018	21-03-2018	51
FM42	2018	11-09-2018	49
FM43	2018	11-09-2018	53

FM44	2018	11-09-2018	52
FM45	2018	11-09-2018	52
FM46	2018	11-09-2018	50
FM47	2018	24-09-2018	68
FM48	2018	25-09-2018	49
FM49	2018	07-10-2018	55
FM50	2018	07-10-2018	50
FM51	2018	07-10-2018	51
FM52	2018	08-10-2018	54
FM53	2018	08-10-2018	54
FM54	2018	10-10-2018	54
FM55	2018	10-10-2018	53
FM56	2018	10-10-2018	49
FM57	2018	10-10-2018	55
FM58	2018	10-10-2018	49
FM59	2018	10-10-2018	55
FM60	2018	16-10-2018	52
FM61	2018	16-10-2018	48
FM62	2018	16-10-2018	54
FM63	2018	19-10-2018	50
FF1	2018	16-03-2018	43
FF2	2018	16-03-2018	46
FF3	2018	19-03-2018	46
FF4	2018	21-03-2018	43
FF5	2018	21-03-2018	44
FF6	2018	21-03-2018	44
FF7	2018	21-03-2018	44
FF8	2018	21-03-2018	43
FF9	2018	21-03-2018	45
FF10	2018	21-03-2018	42
FF11	2018	21-03-2018	43
FF12	2018	21-03-2018	41
FF13	2018	21-03-2018	41
FF14	2018	21-03-2018	44
FF15	2018	21-03-2018	43
FF16	2018	21-03-2018	44
FF17	2018	21-03-2018	45
FF18	2018	21-03-2018	44
FF19	2018	21-03-2018	42
FF20	2018	21-03-2018	45
FF21	2018	21-03-2018	43
FF22	2018	21-03-2018	44
FF23	2018	21-03-2018	45
FF24	2018	21-03-2018	44
FF25	2018	21-03-2018	44
FF26	2018	21-03-2018	44
FF27	2018	21-03-2018	43
FF28	2018	21-03-2018	44
FF29	2018	21-03-2018	45
FF30	2018	21-03-2018	46
FF31	2018	21-03-2018	45
FF32	2018	21-03-2018	44
FF33	2018	21-03-2018	44
FF34	2018	21-03-2018	46
FF35	2018	21-03-2018	44
FF36	2018	21-03-2018	45
FF37	2018	21-03-2018	43
FF38	2018	21-03-2018	45
FF39	2018	21-03-2018	43

FF40	2018	21-03-2018	44
FF41	2018	21-03-2018	44
FF42	2018	21-03-2018	42
FF43	2018	21-03-2018	44
FF44	2018	21-03-2018	43
FF45	2018	21-03-2018	44
FF46	2018	21-03-2018	43
FF47	2018	21-03-2018	41
FF48	2018	21-03-2018	43
FF49	2018	21-03-2018	45
FF50	2018	21-03-2018	40
FF51	2018	21-03-2018	42
FF52	2018	21-03-2018	43
FF53	2018	21-03-2018	45
FF54	2018	21-03-2018	43
FF55	2018	21-03-2018	45
FF56	2018	21-03-2018	43
FF57	2018	21-03-2018	45
FF58	2018	21-03-2018	43
FF59	2018	21-03-2018	44
FF60	2018	21-03-2018	41
FF61	2018	21-03-2018	43
FF62	2018	21-03-2018	43
FF63	2018	24-09-2018	43
FF64	2018	24-09-2018	43
FF65	2018	25-09-2018	47
FF66	2018	07-10-2018	44
FF67	2018	07-10-2018	48
FF68	2018	08-10-2018	45
FF69	2018	10-10-2018	44
FF70	2018	10-10-2018	45
FF71	2018	10-10-2018	45
FF72	2018	10-10-2018	41
FF73	2018	16-10-2018	45
FF74	2018	16-10-2018	46
FF75	2018	16-10-2018	44
FF76	2018	16-10-2018	44
FF77	2018	16-10-2018	47
FF78	2018	18-10-2018	46
FF79	2018	18-10-2018	45
FF80	2018	18-10-2018	43
FF81	2018	19-10-2018	46
FF82	2018	19-10-2018	45
FF83	2018	01-11-2018	47
FF84	2018	01-11-2018	50
